# Home treatment with conestat alfa in attacks of hereditary angioedema due to C1-inhibitor deficiency

**DOI:** 10.1186/1939-4551-8-S1-A23

**Published:** 2015-04-08

**Authors:** Henriette Farkas, Nora Veszeli, Erika Szabo, Dorottya Csuka, Zsuzsanna Zotter, Lilian Varga

**Affiliations:** 1 Semmelweis University, Hungary

## Background

Conestat alfa, a recombinant C1-inhibitor concentrate (rhC1-INH), is a novel therapeutic option for the acute treatment of hereditary angioedema due to C1-inhibitor deficiency (C1-INH-HAE).

## Methods

We analyzed 137 edematous episodes requiring acute treatment and occurring in 6 C1-INH-HAE patients. The patients were treated at home with a dose of 2100 U rhC1-INH per occasion. They recorded the time of rhC1-INH administration, time until the symptoms stopped worsening, time to the onset of symptom relief and to the complete resolution of symptoms. Any side effects were recorded in addition. Symptom severity and patient satisfaction were measured with a visual analogue scale (VAS).

## Results

70 HAE attacks occurred in abdominal viscera, 4 in the upper airways, 35 in subcutaneous, and 28 in multiple locations. RhC1-INH was administered 60.0 (0.0-990.0) [median (min-max)] minutes after the onset of the attacks with a severity (upon injecting) of 57.0 (10.0-99.0) on a VAS. Clinical symptoms improved within 40.0 (0.0-900.0) minutes, and their complete resolution took 600.0 (88.0-3525.0) minutes. The time between the onset of the attack and the administration of rhC1-INH correlated with the time until the symptoms stopped worsening (R=0.3489, p<0.0001), time to the onset of symptom relief (R=0.2492, p=0.0041) and time to the complete resolution of symptoms (R=0.4541, p<0.0001). A second injection of rhC1-INH was administered in 5 attacks, because the symptoms did not improve or resolve completely.

None of the patients experienced a recurrence of the attack, or drug-related systemic adverse events. The mean VAS score of patient satisfaction was 95.8.

## Conclusions

Home treatment with rhC1-INH was an effective and well-tolerated therapy for all types of HAE attacks. Early treatment of the attacks resulted in better outcomes.

